# The Relationship Between Socioeconomic Status and Scalp Event-Related Potentials: A Systematic Review

**DOI:** 10.3389/fnhum.2021.601489

**Published:** 2021-01-27

**Authors:** Hiran Perera-W.A., Khazriyati Salehuddin, Rozainee Khairudin, Alexandre Schaefer

**Affiliations:** ^1^Faculty of Social Sciences and Humanities, Universiti Kebangsaan Malaysia, Bangi, Malaysia; ^2^Jeffrey Cheah School of Medicine and Health Sciences, Department of Psychology, Monash University Malaysia, Subang Jaya, Malaysia

**Keywords:** socioeconomic status, SES, poverty, event related potentials, slow waves, executive function, cognitive control, attention

## Abstract

Several decades of behavioral research have established that variations in socioeconomic status (SES) are related to differences in cognitive performance. Neuroimaging and psychophysiological techniques have recently emerged as a method of choice to better understand the neurobiological processes underlying this phenomenon. Here we present a systematic review of a particular sub-domain of this field. Specifically, we used the PICOS approach to review studies investigating potential relationships between SES and scalp event-related brain potentials (ERP). This review found evidence that SES is related to amplitude variations in a diverse range of ERPs: P1, N1, N2, Error-Related Negativities (ERN), N400, auditory evoked potentials, negative difference waves (*Nd*), P3 and slow waves (SW). These ERPs include early, mid-latency and late potentials that reflect a broad range of cognitive processes (e.g., automatic attentional processes, overt attention, language, executive function, etc.). In this review, all SES effects on ERPs appeared to reflect an impairment or a less efficient form of task-related neural activity for low-SES compared to high-SES individuals. Overall, these results confirm that a wide variety of distinct neural processes with different functional meanings are sensitive to SES differences. The findings of this review also suggest that the relationship between SES and some ERP components may depend on the developmental stage of study participants. Results are further discussed in terms of the current limitations of this field and future avenues of research.

## Introduction

The psychological and neural correlates of socioeconomic inequalities have become a topic of renewed interest in recent years. In particular, a growing number of studies have shown that people growing up in socially disadvantaged environments have a weaker performance in a wide range of cognitive tests (Raizada and Kishiyama, 2010; Farah, 2017). Although earlier results pointed toward similar directions (e.g., Kornhauser, 1918), the current renewal of interest for this topic is driven by the widely accepted notion that understanding the relationship between socioeconomic variables and cognitive function could eventually lead to policies aimed at tackling the poverty cycle (Haushofer and Fehr, 2014). The current interest for this field of research is also motivated by the existence of novel theoretical and methodological tools that enable a more fine-grained understanding of how socioeconomic condition can relate to the human mind. In this article we present a systematic review of a specific subdomain of this research field, as we examined studies investigating how socioeconomic status (SES) predicts changes in brain activity measured with brain event-related potentials (ERP), a widely used method in neuroscience.

Hereafter, we first briefly discuss the concept of SES and how it is measured. Next, we briefly summarize the current state of the wider research field focusing on the relationship between SES and cognitive function and how EEG/ERP studies can contribute to it. Next, we describe the methods and results of our systematic review. It is important to highlight that the specific scope of the following section is to explain how EEG/ERP studies can contribute to understanding the relationship between SES and cognition. Therefore, the goal of this section is not to provide an exhaustive review of how SES relates to cognitive function in general, a topic that has often been extensively reviewed by others (Evans and Kim, 2007; Duncan et al., 2012; Kim et al., 2013; Farah, 2017, 2018).

### Definitions and Operationalization of Socioeconomic Status

In order to understand the topic of how socioeconomic factors relate to cognition, it is important to clarify what is understood by *socioeconomic status* (SES). SES is a concept referring to the actual or perceived position of an individual or a group in a given social context. It has different components that can refer to different measurement methods. For instance, Duncan and Magnuson (2012) have proposed a distinction between economic, educational, and occupational components of SES, which overlaps with common operationalizations of SES in social sciences (Galobardes et al., 2006). The economic component refers to material resources (income, assets, and financial resources), the educational component refers to the level of education attained by an individual or their parents; and the occupational component often refers to the complexity of an individual's occupation. Occupational complexity in this context typically refers to the intellectual demands of a profession, and it can be measured by country-specific complexity rankings (Smart et al., 2014).

Subjective self-assessment of SES is also a common facet of SES, which involves asking individuals to self-rate their economic situation or their relative position in the society (e.g., Adler et al., 2000). In the literature focusing on how SES relates to cognition, income-based estimates of SES are often prioritized (per capita income, household^1^ income, income-to-needs, etc.). Asset-based measures of SES are also used. For instance, one of the earliest studies on the psychological correlates of SES found that children living in households owning telephone sets at the beginning of the twentieth century had better academic outcomes than children in households who didn't (Kornhauser, 1918). It has been suggested that asset-based measures can often capture dimensions of people's economic lives that can be missed by income measures (Brandolini et al., 2010). For instance, a person without a regular income may have an easy access to resources if they own large financial or material assets. Beyond the concept of SES, the concept of “poverty” is also sometimes used. It is often related to specific criteria (e.g., income thresholds) indicating that an individual or a group is living in difficult or substandard conditions (Duncan et al., 2012; The World Bank, 2020).

There is evidence of poorer cognitive performance for individuals with low compared to high levels of SES across a wide range of different SES indicators. For instance, this effect was observed for differences in income (e.g., Tine, 2014; Hackman et al., 2015), assets (Kornhauser, 1918; Fernald et al., 2011), subjective SES (Loeb and Hurd, 2019), occupational complexity (Farah et al., 2006), parental education (Kaplan et al., 2001; Fernald et al., 2011). However, we detailed in **Table 2** which specific method of SES assessment has been utilized by each of the EEG/ERP studies that we reviewed. For the sake of conciseness, we will use the generic terms of low-SES and high-SES to refer to different but commonly accepted indicators of SES including the ones referred to above.

### SES and Cognition

Most contemporary research on how SES relates to cognition has reported findings suggesting that low-SES individuals^2^ present performance deficits in behavioral tasks assessing cognitive processes. For instance, Hoff-Ginsberg (1998) has found that high-SES children had a more advanced lexical development than low-SES children, and Fernald et al. (2013) have shown significant disadvantages in language development for low-SES children. These two studies are instances of a more general trend showing that SES predicts language performance (Raizada and Kishiyama, 2010).

Other reports from a relationship between SES and cognitive performance come from a large body of research showing that low-SES individuals have lower performance than high-SES in tasks assessing Executive Function (EF), or “Cognitive Control.” EF refers to a set of higher-order cognitive processes thought to be essential to goal-directed behaviors. EF processes rely on a frontoparietal network, and they are implemented when automatic schemata are not sufficient to attain a task goal (Mushtaq et al., 2011). They can include cognitive inhibition processes, working memory updating, set-shifting, active maintenance of information in working memory and controlled retrieval from long-term memory (Miyake et al., 2000; Baddeley, 2006; Braver et al., 2007; Ruge and Braver, 2007; Friedman and Miyake, 2017). A substantial amount of evidence indicates that, on average, low-SES individuals perform less well than high-SES people in EF tasks such as working memory (WM), inhibition, planning and executive attention (Mezzacappa, 2004; Noble et al., 2006; Hackman and Farah, 2009; Farah, 2018). The majority of these studies have tested children, but studies on adults have also shown deficits in EF tasks for low-SES individuals (e.g., Mani et al., 2013).

There is also an extensive literature showing that low-SES children have poorer academic outcomes than high-SES children (McLoyd, 1998; Roy et al., 2014; Hair et al., 2015). In addition, studies have also shown that SES predicts different patterns of decision-making behaviors, as evidence shows that low-SES people are more risk-averse and less willing to delay rewards than high-SES individuals in financial decision-making tasks (Ong et al., 2019).

These results raise the issue of whether SES has specific or general effects on cognitive functioning. A general effect would imply that SES has a uniform “blanket” effect on a variety of different cognitive processes. This possibility could occur if SES has an effect on neural processes common to a large variety of cognitive functions (e.g., if SES modulated cortical development). Specific effects of SES would imply that some cognitive process(es) would be more vulnerable to SES effects than others. For instance, EF processes could potentially be thought to be more vulnerable to SES effects, given existing evidence indicating that EF is involved in many of the tasks commonly correlated with SES (Farah, 2017; Lawson et al., 2018).

Research using neuroimaging techniques (both functional and structural MRI) has revealed that SES is related to both structural differences and differences in task-related functional brain activity. For instance, Mackey et al. (2015) have found that low-SES students had on average smaller cortical gray matter volume than high-SES students, and this finding was consistent across all lobes of the brain. Several other studies found similar brain volume reductions in low-SES individuals (e.g., Noble et al., 2015; Rosen et al., 2018; Leonard et al., 2019). Studies have found that low-SES is associated with smaller hippocampal volume (Noble et al., 2015). SES differences were also reported in functional brain activity studies using fMRI. For instance, Rosen et al. (2018) found higher levels of brain activity in prefrontal areas during a WM task for high-SES compared to low-SES children. Functional differences were also found in tasks not directly related to EF, such as language-related tasks (Hackman et al., 2010; Raizada and Kishiyama, 2010; Perkins et al., 2013).

The multiplicity of brain areas related to SES differences and evidence suggesting that SES is related to a whole-brain reduction in gray matter volume (Mackey et al., 2015) may suggest a general effect of SES on cognitive function. However, frequent reports of SES differences in neural systems related to EF may favor the hypothesis that EF is particularly vulnerable to SES. Recent research has moved toward testing explanative models of why SES relates to cognitive function. These models see SES as a construct reflecting variations in exposure to factors that could have a direct effect on cognitive function throughout an individual's life. These factors could include the amount and quality of cognitive stimulation throughout someone's life (Bradley et al., 2001; Rowe and Goldin-Meadow, 2009; Amso et al., 2018; Last et al., 2018; Rosen et al., 2018, 2019), cumulative stress (Evans and Schamberg, 2009; Kim et al., 2013) and frequent worries about material scarcity (Shah et al., 2012; Mani et al., 2013; although see Wicherts and Scholten, 2013).

In summary, extant research has shown that, on average, low-SES individuals perform more poorly than high-SES people on a series of cognitive tasks, chiefly amongst them tasks related to EF. The current state of the literature suggests that some functions are clearly related to SES differences (EF and language). However, it is still unclear if SES has a general “blanket” effect on a wide variety of different cognitive processes. Neuroimaging research gives some credence to the possibility of SES effects on a common neural factor (i.e., gray matter thickness across different brain lobes, Mackey et al., 2015). However, the issue of whether some cognitive functions (e.g., EF) might be more vulnerable to SES effects than others is still a matter of debate.

### Using Event-Related Potentials to Investigate the Relationship Between SES and Cognition

The event-related potentials method can contribute to provide answers to some of these outstanding questions. ERPs are obtained through averaging EEG activity time-locked to categories of specific stimuli or behavioral responses. This process of averaging isolates ERP components, or ERP effects, which reflect brain electrical activity produced by the synchronous firing of large groups of neurons captured by scalp electrodes. Several ERP components have been identified, and a vast body of research built upon 60 years has enabled researchers to link different ERP components to specific cognitive processes in such a way that ERPs are now often used as biomarkers of cognitive processes (Luck, 2005; Pavlov et al., 2020). It is not within the scope of the present article to review the functional meaning of different ERPs (we refer to existing authoritative publications on this topic, e.g., Luck, 2005; Luck and Kappenman, 2011). However, we describe the spatiotemporal and functional properties of the main ERPs tested by the studies of this review in the Results section. ERPs have a very high temporal resolution, which enables researchers to categorize them according to their timing. Typically, several early ERPs (e.g., P1, N1, and P2) are often seen as reflecting automatic or “obligatory” processes such as automatic attentional orientation processes (Schupp et al., 2006b; Carretié et al., 2008; Walker et al., 2011; Yong et al., 2020). A number of ERPs occurring later in the processing stream are often seen as reflecting overt and controlled processes (Schupp et al., 2006a) often linked to EF processes. For instance, the parietal P3b is linked to overt recognition and working memory updating processes and late slow waves to sustained maintenance of information in WM (Ruchkin et al., 1988; Revonsuo and Laine, 1996; García-Larrea and Cézanne-Bert, 1998; Polich, 2007; Watts et al., 2014; Bailey et al., 2016). Mid-latency ERPs, such as the N400 are often linked to familiarity and other recognition processes (Yong et al., 2020).

These properties of ERP components could also allow us to examine some of the outstanding questions in the relationship between SES and cognitive processes. First, the weight of available evidence linking a large variety of ERP components to specific cognitive processes could help to map which specific cognitive processes are most vulnerable to SES effects. A corollary to this possibility is that ERPs could contribute to estimating how general is the relationship between SES and neural correlates of cognitive processes. Second, ERPs could contribute to testing whether EF is specifically sensitive to SES effects. If this possibility is true, then ERPs typically linked to EF (e.g., slow waves and P3b related to EF tasks) would be affected by SES, whereas ERPs linked to processes thought to be dissociated from EF (e.g., early automatic processes) would not.

In order to examine these questions, we have used the PICOS approach to systematically review studies which used brain event-related potentials to compare high-and low-SES individuals.

## Methods

This systematic review followed the PRISMA (Preferred Reporting Items for Systematic reviews and Meta-analysis) guidelines (Moher et al., 2010).

### Eligibility Criteria

This systematic review followed the PICOS approach (Tacconelli, 2010) to formulate the following research question: what evidence is available that identifies changes in brain activity (Outcome; O) in those who are in low-SES (Population; P) compared to those who are in high-SES (Comparison; C)? This systematic review is limited to quasi-experimental or experimental studies comparing low-SES and high-SES groups (Study design; S) that used the ERP method (Intervention; I) to explore this research question.

Experimental studies should have the following criteria to be eligible in this systematic review: (1) the sample should include participants from low-SES and high-SES groups either in the form of specific group comparisons or in the form of a continuous SES variable diverse enough to contain both high and low-SES individuals; (2) the studies should employ the ERP technique. Studies that looked at changes in EEG oscillations were not included as they are beyond this review's scope; (3) In addition, we wanted to focus on studies that measured ERPs from people who truly live in high or low-SES conditions in their everyday lives. Therefore, we did not include studies that manipulated transient monetary gains and losses in the context of temporally limited experiments; (4) Further, given that psychopathological disorders can modulate ERP effects (e.g., Ruchensky et al., 2020), we focused on studies that tested non-clinical samples.

### Information Sources and Search Strategy

We performed a comprehensive literature search in PubMed, Web of Science (WoS) databases. We also used Google Scholar to identify possible additional articles that corresponded to our criteria. We found no articles in Google Scholar which we hadn't yet identified through the other databases. Reference lists of selected articles were further explored to identify additional relevant studies. Studies were limited to peer-reviewed articles and those published in the English language. When necessary, additional information was requested to the original authors. Search results included articles from January 1990 to February 2020, which covered 20 years of research. This time period was selected because we could not find SES-related ERP studies prior to 1990 that matched our inclusion criteria. The electronic databases were searched again in the 3rd week of October 2020 to identify the most recent studies.

In our criteria, we accepted SES to be operationalized in a broad sense compatible with the different SES components and methods of measurements commonly associated with this construct. We used poverty and scarcity related search terms such as “monetary scarcity,” “lack of money,” “lack of basic needs,” and “lack of resources” in our search. We also searched SES-related keywords such as “poor,” and “poverty,” “low-SES,” and “socioeconomic status.” These search terms unveiled studies that used a relatively broad range of methods to operationalize SES. These methods are overall compatible with SES indicators often used in the literature (income, assets, education, occupation, etc.). The search terms also had to co-occur with the following keywords related to ERP methods such as “electroencephalography,” “ERP,” “evoked potentials,” and “event-related potentials.” Only studies that involved human participants were included in this systematic review. Studies in which ERPs were combined with fMRI techniques were considered as acceptable in our criteria.

These criteria produced a vast number of search results, which were narrowed through inclusion and exclusion criteria detailed in Table 1. For studies mentioning scarcity, we carefully checked whether this concept was truly related to socioeconomic status. We included studies that involved both adults and children.

**Table 1:** Inclusion and Exclusion criteria.

**Inclusion criteria**	**Exclusion criteria**
***Study design***	
• Experimental studies	• Reviews, meta-analyses, letters, editorials, conference proceedings
***Population***	
• All age groups • Income • High or low SES living conditions	• Animals • Clinical samples (e.g., Psychopathological disorders and other health issues) • Traumatic brain injuries
***Topic***	
• Poverty • Socioeconomic status (SES) • Event-related potentials (ERP)	• EEG oscillations • Scarcity manipulations that only involve transient and small changes in monetary gains and losses

Studies on clinical samples that looked at addiction, brain injuries, neurobiological disorders, psychopathological disorders or other medical problems were not included in this systematic review. We also excluded all forms of review articles. However, the reference lists in those review studies were carefully screened to identify potentially relevant studies.

### Study Selection

Studies were selected based on predefined inclusion and exclusion criteria in terms of the methods used to gather data, population, and the topic of the study. Studies were excluded if they did not match our criteria, on the basis of information available in the abstract. However, if the abstract of a study did not provide enough information, then the full text was carefully examined before deciding on the inclusion or exclusion of this study (See Table 1 for inclusion/Exclusion criteria).

**Figure 1:**
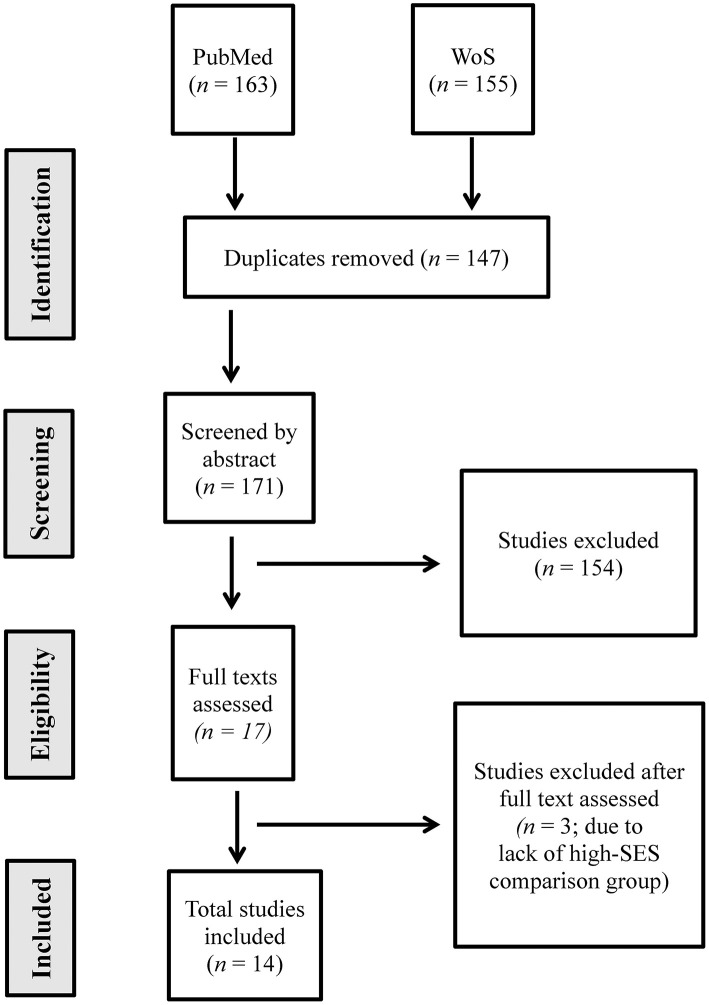
PRISMA flow chart showing the process of article screening and selection process for this systematic review (*n* = number).

The first (HP-W.A.) and the second (KS) authors independently selected the articles from the titles and abstracts for subsequent full-texts and compared the results. In case of a disagreement, a third opinion was provided by the third (RK), or the fourth (AS) authors to solve any disputes. The authors maintained an inter-observer agreement and resolved any disagreements via discussion until a consensus was reached during the article selection phase. Figure 1 indicates the selection process in detail.

### Data Collection Process and Data Items

Based on the included studies, the following information was extracted (see Table 2): (1) population (SES group category, i.e., low-SES or high-SES, sample sizes, mean age, and gender); (2) study design and type of behavioral task used; (3) how SES was operationalized; (4) the primary EEG-ERP results. ERP waveforms are summarized in Table 3. Reference electrodes and numbers of artifact-free trials are presented in Supplementary Table 1.

**Table 2:** Data items.

**References**	**Sample size (*****n*****)** **(Mean Age: y)**		**Gender** **Male (Female)**	**Experimental task**	**SES operationalization**	**Results**
	**Low-SES**	**High-SES**				
Katus et al. (2020)	*n* = 221 (1-month) *n* = 210 (5-months)	*n* = 50 (1-month) *n* = 53 (5-months)	264 (270)	Habituation-novelty task with auditory stimuli of three different categories: frequent, infrequent, and trial unique sounds.	**High-SES:** UK cohort families lived within the 20-miles radius within the university premises belonging to both urban and rural communities. **Low-SES:** Gambian cohort families are from Mandinka ethnic group who lived in or near surrounding villages near rural West Kiang district in Gambia.	No differences in early components (N1, P1, N2) were observed in both cohorts. High-SES had a high P3 amplitude compared to Low-SES. Latency was reduced with age from 1- to 5-months old.
Ralph et al. (2020)	*n* = 30 (9.03)	*n* = 34 (8.91)	30 (34)	Word learning from context task	**High-SES:** not eligible for a free meal. **Low-SES**: those who are qualified for free lunch or meals at a reduced rate as a proxy for income and maternal education that predicts the low-SES status.	No SES differences were found to known words. When learning new words, High-SES showed a significant N400 amplitude attenuation when learning a new word. This effect was not found for the Low-SES.
Wray et al. (2017)	*n* = 44 (4.3)	*n* = 14(4.3)	16 (42)	Dichotic listening task	**High-SES:** who are living above the national poverty line. **Low-SES:** those who are living at or below the national poverty line (threshold for a family of four).	At age 4, High-SES exhibited higher ERP amplitude for the attended stimuli compared to the Low-SES. At age 5, Low-SES showed a similar high amplitude effect as the age 4 high-SES children with reduced distractor suppression effect compared to the High-SES.
Kishiyama et al. (2009)	*n* = 13 (9.5)	*n* = 13 (9.5)	6 (20)	Stimuli-target discrimination task	SES measure was based on the MacArthur-Sociodemographic Questionnaire; based on primary caregiver education, average household income, and income-to-needs ratio. **High-SES:** Parents has a bachelor's degree, mean income $96,157, income to needs ratio 4.87 **Low-SES:** parents do not have a bachelor's degree, mean household income was $27,192, income-to-needs ratio was 1.0	Low-SES had a reduced early extrastriate (P1, N1) and novelty (N2) ERP amplitudes compared to the High-SES.
D'Angiulli et al. (2008)	*n* = 12 (13.8)	*n* = 16 (12.7)	13 (15)	Auditory selective attention task	SES scores were computed using Hollingshead four-factor index of social status (Hollingshead, 1975), residential area quality, parental education, and occupation. **High-SES:** parents are college graduates and hold managerial positions **Low-SES:** parents are high-school graduates and skilled workers.	High-SES children showed a larger Nd difference effect compared to Low-SES children for the attended and unattended stimuli.
D'Angiulli et al. (2012)	*n* = 14 (12.9)	*n* = 14 (13.7)	11 (17)	Auditory selective attention task	SES score was computed using Hollingshead (1975) four-factor index of social status (1975); also based on parental occupation, education, household income. **High-SES:** parents are college graduates and hold managerial positions **Low-SES:** parents are high-school graduates and skilled workers.	High-SES showed greater ERP Nd difference for attended and unattended stimuli compared to the Low- SES.
St. John et al. (2019)	*n* = 26 (5.5)	*n* = 43 (5.5)	29 (40)	Visual Go/No-go task	**High-SES:** income more than the national poverty line **Low-SES:** based on income-to- needs ratio of 3.0 (income less than three times the federal poverty line based on the household size).	High-SES had a larger P3b amplitude for both go and no-go trials compared to the Low-SES.
Ruberry et al. (2017)	*n* = 76 (36–40 months)	*n* = 42 (36–40 months)	59 (59)	Flanker task, Frog/fish task	**High-SES:** annual income above national poverty line **Low-SES:** those who are at or below 1.5 times federal poverty line for annual income.	Income was not related to ERP measures. No neural activity differences were found for executive control and executive attention between Low and High-SES groups.
Giuliano et al. (2018)	*n* = 71 (4.3)	*n* = 33 (4.3)	54 (50)	Auditory selective task	**High-SES:** better maternal education, INR more than the national poverty line. **Low-SES:** (1 or more socioeconomic risks) was based on low maternal education, single parenthood, low household income (based on income-to-needs ratio of 0.5 below the national poverty line).	Low-SES showed a larger amplitude for distractor sounds compared to the High-SES.
Skoe et al. (2013)	*n* = 33 (14.52)	*n* = 33 (14.58)	36 (30)	Auditory brain response (ABR) paradigm	Maternal education was used as a proxy for SES. **High-SES:** Mothers who completed post-secondary schooling **Low-SES:** Mothers had no post-secondary schooling.	Low-SES had a noisier, weaker, variable auditory ERP response compared to the High-SES.
Czernochowski et al. (2008)	*n* = 7 (73.86)	*n* = 7 (73.86)	(14)	Recency memory task	Hollingshead (1975) two-factor variation of the index of social position was used to assess SES category, SES value was subtracted from 100. **High-SES:** corresponded to high scores. **Low-SES:** corresponded to low scores.	High-SES showed a larger SW effect for correct recency memory trials compared to Low-SES.
^*^Conejero et al. (2016)	*n* = 52 (16.7 months) *n* = 14 (21.9 mean age) In this study, SES was a continuous variable.		26 (26) 1 (13)	Three-piece puzzle formation task either correctly or incorrectly	Parental education (rated from 1–no studies to 7- postgraduate studies), income-to-needs ratio, and parental occupations were used to assess the SES status.	Low-SES was associated with reduced ERN amplitude compared to the High-SES.
^**^Brooker (2018)	*n* = 119 (3.9 mean age) In this study, SES was a continuous variable (e.g., Annual household income ranged from 15k to >90k)		50 (69)	Modified go/no-go task (spaceship—no-go/asteroid—go)	Parent's annual income, Parental education, Hollingshead four-factor index of social status (Hollingshead, 1975).	No ERN difference between the SES groups. But greater ERN amplitude at age 3 predicted a similar pattern at age 4 in high-SES which was also associated with high parental sensitivity. This pattern was not observed in Low-SES.
Wang and Yang (2020)	*n* = 68 (19.5)	*n* = 67 (19.5)	*Gender not stated*	Response selection/inhibition task based on threat and non-thread words.	**High-SES:** income per capita more than the urban **Low-SES:** income per capita < ¥1100 in rural, and ¥2250 in urban	High-SES had a larger P3 amplitude and long latency compared to the Low-SES group.

* *Exact number of low and high-SES participants were not mentioned. SES was included as a continuous scale of measurement ranging from low to high*.

** *Approximately 14.14% of participants are from low-SES. The remaining participants (85.85%) belonged to middle to high-SES income categories*.

**Table 3:** ERP summary.

**References**	**ERP**	**Measure**
Katus et al. (2020)	N1 P1 N2 P3	Mean amplitude and latency Mean amplitude and latency Mean amplitude and latency Mean amplitude and latency
Ralph et al. (2020)	N400	Mean amplitude
Wray et al. (2017)	Difference negativity (*Nd*)	Mean amplitude
Kishiyama et al. (2009)	P1 N1 P2 N2 P3 Slow wave (SW)	Peak amplitude Peak amplitude Peak amplitude Peak amplitude Peak amplitude Peak amplitude
D'Angiulli et al. (2008)	Difference negativity (*Nd*)	Mean amplitude and latency
D'Angiulli et al. (2012)	Difference negativity (*Nd*)	Mean amplitude and latency
St. John et al. (2019)	P3	Mean amplitude
Ruberry et al. (2017)	N2 P3	Mean amplitude Mean amplitude
Giuliano et al. (2018)	Difference negativity (*Nd*)	Mean amplitude
Skoe et al. (2013)	Auditory sensory potentials	Mean squared amplitude
Czernochowski et al. (2008)	Slow wave (SW)	Mean amplitude
Conejero et al. (2016)	ERN	Mean amplitude
Brooker (2018)	ERN	Mean amplitude
Wang and Yang (2020)	P3	Mean amplitude and latency

## Results

**Figure 2:**
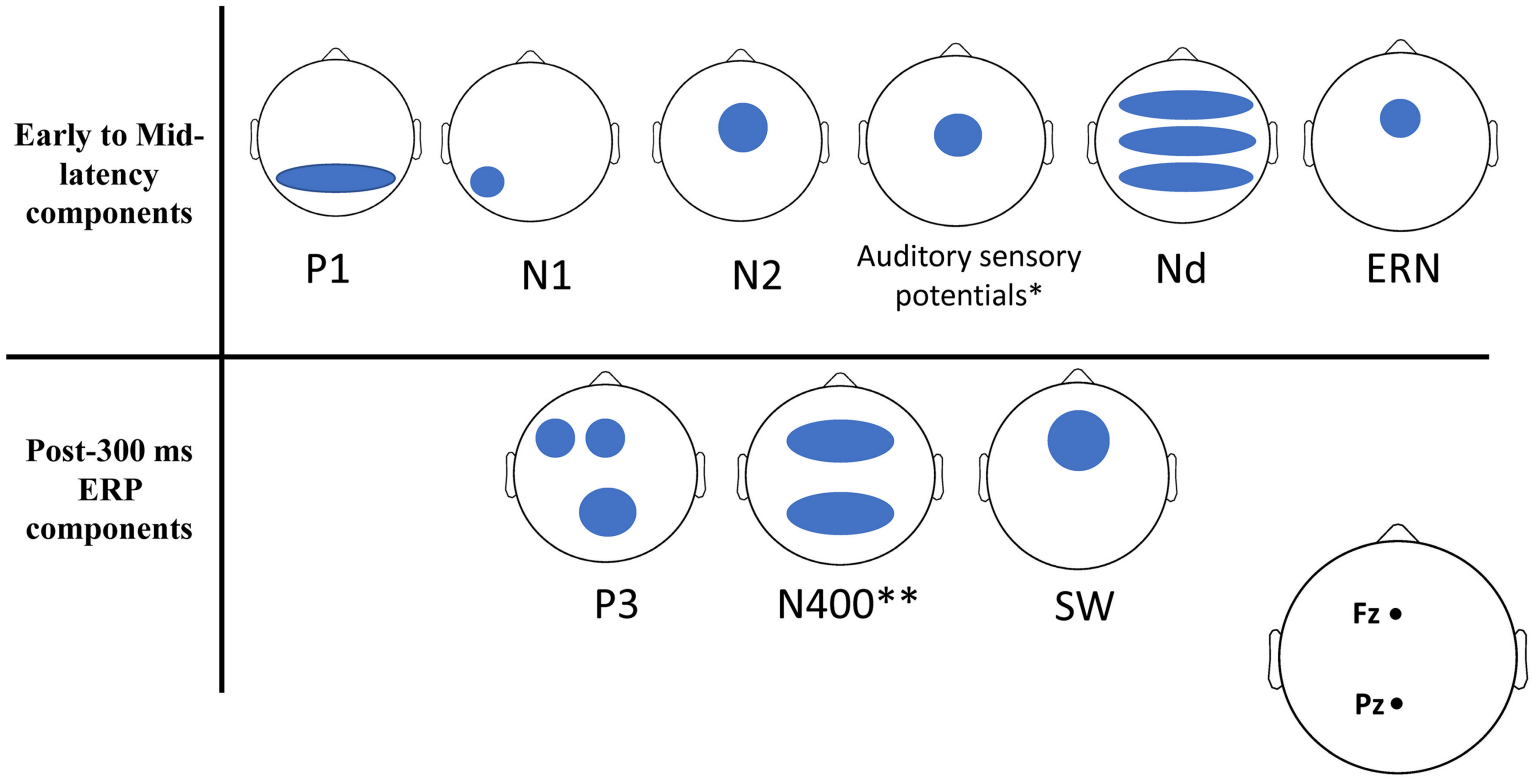
Schematic scalp locations of ERP components correlated with SES. Here we schematically represented on 2D scalp maps the approximate topography of each ERP component for which a significant relationship with SES was found in any of the studies examined by this review. For any given ERP component, each blue shape represents an approximate scalp location where it correlated with SES in at least one of the studies of this review.

A total of 14 studies were included in this systematic review. The selected studies consist of a total of 1,429 participants (more than 800 are from low-SES groups). All selected participants were healthy and did not have any history of severe chronic mental disorders, traumatic brain injuries, alcohol abuse, or developmental disorders. We hereafter list the ERP components that were investigated by the studies included in this review. We provide a brief description of the spatio-temporal and functional properties of each component and we next outline how these components were related to SES in the selected studies. Figure 2 provides a description of the ERPs that were associated with SES, and a detailed summary of the findings can be found in Table 3.

### Early to Mid-latency Components

Some of the studies selected by this review have examined ERP effects in early to mid-latency components, which can be defined as ERPs occurring approximately before 300 or 400 ms post-stimulus onset (Luck, 2005; Olofsson et al., 2008; Watts et al., 2014; Goto et al., 2017). These components often reflect automatic or obligatory processes (e.g., automatic orientation processes). However, some of them can be modulated by top-down processes and some components (e.g., N2 and ERN) can reflect processes that are antecedents of cognitive control processes (Yeung et al., 2004).

#### P1

The P1 component is an early positive peak typically observed at occipital sites between ~100 and 130 ms post-stimulus onset and it reflects early selective attentional processes (Yago et al., 2004). Amongst studies selected in this systematic review, Kishiyama et al. (2009) reported a reduced P1 component in low-SES compared to high-SES children aged 9.5 years old. However, another study reported an absence of SES effects on P1 amplitude in 1- or 5-month-old infants (Katus et al., 2020). It has to be noted that Katus et al. (2020) used a frontal (Fz) electrode to quantify the P1, whereas Kishiyama et al. (2020) used more typical posterior locations.

#### N1

The N1 is an early negativity peaking around 70–200 ms post-stimulus onset and it can be found in both anterior and posterior electrode sites. The N1 has been linked to stimulus discrimination and detection tasks (Hillyard et al., 1973), and a variety of early attentional processes including stimulus expectancy (Picton and Hillyard, 1974; Starr et al., 1997). In this review, Katus et al. (2020) found no SES effects on N1 amplitude using a sample of 1- and 5-month-old infants. However, Kishiyama et al. (2009) reported reduced N1 amplitudes in low-SES compared to high-SES children older than the ones tested by Katus et al. (mean age of 9.5 years). Similarly to the P1, Kishiyama et al. (2009) quantified the N1 from posterior locations, whereas Katus et al. (2020) used a midfrontal electrode (Fz).

#### P2

The P2 is an early positivity following the N1 component. It tends to be larger in frontal sites, but it can also be observed in posterior locations and it typically peaks between 100 and 250 ms post-stimulus onset (Yong et al., 2020). It is often associated to a rapid allocation of attentional resources which can be mobilized for a variety of task goals such as the perception of threatening stimuli or successful memory encoding processes (Carretié et al., 2008; Olofsson et al., 2008; Schaefer et al., 2011). Reduced P2 amplitude, can also be associated with learning difficulties in reading, writing, and arithmetic domains (Fernández et al., 2014). In the current review, Kishiyama et al. (2009) found no significant relationship between SES and P2 amplitude.

#### N2

The N2 is a negative peak occurring after the P2, which can be observed approximately between 200 and 400 ms post stimulus onset on frontal-central sites (Folstein and Van Petten, 2008, Goto et al., 2017). The N2 is thought to reflect early attentional processes toward motivationally or task-relevant information (Olofsson et al., 2008; Walker et al., 2011; Goto et al., 2017). In the context of EF-related tasks, the N2 is thought to reflect cognitive conflict, an antecedent of EF processes (Yeung et al., 2004; Heidlmayr et al., 2020). In this systematic review, Ruberry et al. (2017), found no relationship between SES and N2 in a study focusing on 36–40 months old infants. Similarly, Katus et al. (2020) failed to observe SES effects on N2 amplitude for 1–5 months old infants. Interestingly, however, Kishiyama et al. (2009), found reduced N2 amplitude in low-SES compared to high-SES children who were older than those tested in the two studies mentioned above (9.5 years old).

#### Auditory Sensory Responses

Auditory evoked brain potentials can be measured on a very short time window (within 10 to 50 ms post-stimulus onset) from the vertex (Luck, 2005; Skoe and Kraus, 2010). Some of these ERPs can reflect the efficiency of auditory pathways and they can also be modulated by the history of sensory experiences (Skoe et al., 2013). Amongst studies included in this review, Skoe et al. (2013) found noisier auditory evoked responses (within 11.4 and 40.6 ms post-stimulus) among low maternal education individuals (a proxy for low-SES) compared to participants with high maternal education (a proxy for high-SES). Participants were on average 14.5 years-old. Noisier neural activity in this context may reflect a relatively inefficient auditory system (Skoe et al., 2013).

#### Difference Negativity (Nd)

*Difference Negativity, Negative difference wave* (Nd), or *Processing Negativity* (PN) refer to ERP components that reflect voluntary selective attention (Mueller et al., 2008), a process which is often linked to executive function (Shallice, 1988). It involves a paradigm of auditory selective attention in which participants have to attend or ignore specific auditory stimuli. ERPs are time-locked to attended and unattended stimuli, and the Nd/PN effect refers to a significant ERP amplitude difference between these two conditions, which can be quantified with a difference waveform. When isolating individual components of interest, the attended stimuli can elicit larger difference waves compared to the unattended stimuli. For instance, Isbell et al. (2016) reported ERP differences between attended and unattended stimuli between 100 and 300 ms in both central and frontal sites.

In this systematic review, Wray et al. (2017) found in a longitudinal study that high-SES children (aged 3–4 years old) had larger ERP differences between attended and unattended conditions than low-SES children. However, these authors found no SES effects on ERPs related to distractor suppression. One year later, children from the low-SES group (now 4 years old) exhibited an attended-unattended ERP amplitude similar to that previously obtained by high-SES children. However, they also appeared to have a less efficient distractor suppression as ERPs to unattended items was larger compared to the high-SES group. (D'Angiulli et al., 2008, 2012) also found that low-SES children had a smaller Attended-Unattended effect, and D'Angiulli et al. further reported evidence that low-SES individuals allocated more attentional resources to unattended stimuli than high-SES. Using an auditory selective attention method, Giuliano et al. (2018) also found that ERP correlates of distractor sounds elicited larger ERP amplitudes in low-SES participants (Giuliano et al., 2018).

#### Event-Related Negativity (ERN)

The ERN is a waveform time-locked to the commission of an error in a cognitive task (typically a go/No-go task or an Eriksen Flanker task), contrasted to a waveform time-locked to a correct task response. It is a negativity peaking within a period of 100 milliseconds following the task response; it is typically recorded in frontocentral sites and it is linked to activity in the Anterior Cingulate Cortex (ACC). The ERN is thought to reflect the detection of prediction errors but it is also often increasingly seen as a biomarker of psychopathological disorders (Gehring et al., 1993, 2018; Dehaene et al., 1994; Holroyd and Coles, 2002; Van Veen and Carter, 2002; Evans and Kim, 2007; D'Angiulli et al., 2012; Weinberg et al., 2012; Taylor et al., 2018).

In this systematic review, we found that low-SES children (mean age 16.7 months old) exhibited reduced ERN amplitude (Conejero et al., 2016). In addition, Brooker (2018), found that ERN activity was increased in children who were high-SES and who enjoyed high maternal sensitivity. Among the high-SES group, ERN amplitude at age 3 also predicted a similar effect at age 4. However, such an effect was not observed with children from a low-SES background.

### Post-300 ms ERP Effects

#### P3

The P3 ERP component (also known as P300, Late Positive Potential, Late positive Complex) is a positive deflection occurring approximately between 250 and 500 ms following a stimulus (Polich, 2007). It is widely seen as reflecting an overt allocation of attentional resources to a relevant stimulus or task (Donchin, 1981; Polich, 2007). The P3 is often associated to the oddball task, and its amplitude is larger for infrequent stimuli in this task (Luck, 2005). P3 potentials can be divided in two subtypes, the P3a and P3b. The P3b is typically observed in centroparietal sites, and the P3a subtype is most often observed in frontal sites. Although both subtypes reflect differences in attentional allocation, it has been suggested that the P3b is is more stronlgy involved in memory encoding processes (Polich, 2007). Our systematic review unveiled contradictory results regarding the P3. Two studies found no P3 amplitude difference between SES groups (Kishiyama et al., 2009; Ruberry et al., 2017) in samples of children. However, Wang and Yang (2020) found larger P3 amplitude and latency for high-SES compared to low-SES adult participants in a word-matching task. Katus et al. (2020) compared 1–5 months old from a low-income country (Gambia) to a high-income country (UK). They reported increased P3 amplitudes for auditory stimuli on high-SES (UK) compared to low-SES (Gambia) infants. In addition, habituation effects modulated P3 amplitude in the high-SES sample but not the low-SES sample. Similarly, St. John et al. (2019) found that high-SES children at 4.5 to 5.5 years-old show increased P3 amplitudes compared to their low-SES counterparts using a go/no-go task. It is also important to know that Wang and Yang (2020) and Katus et al. (2020) have examined the P3 from frontal sites and St John has obtained effects in posterior sites. Therefore, the former probably reflects the P3a subtype and the latter probably conforms to the P3b.

#### N400

The N400 is a mid-latency negativity peaking between 300 and 400 ms typically observed in central-parietal electrode sites (Kutas and Federmeier, 2011). The centro-parietal N400 component is associated with semantic learning processes, i.e., word learning, language processing, and semantic retrieval (Mestres-Missé et al., 2007; Kutas and Federmeier, 2011). The fontal N400 has been linked to familiarity processes (Eimer, 2000; Yong et al., 2020). Larger N400 amplitude was found during semantic incongruence and during violations of semantic expectations (Luck, 2005; Korpilahti et al., 2017). Reduced N400 amplitude can also reflect successful learning of the meaning of new words. In this context, Ralph et al. (2020) investigated the relationship between SES and the N400 in children from 3 to 5 years of age. Successful learning of new words was associated to a reduction of N400 amplitude. This attenuation of N400 was not observed in low-SES children.

#### Slow Waves

Slow-waves (SW) are sustained ERP deflections observed approximately after 800 ms post-stimulus onset (Ruchkin et al., 1988, 1990). There can be positive or negative slow waves, depending on various task modalities and demands. Typically, SW amplitude increases with working memory demands, and thus they are thought to reflect sustained maintenance in working memory (Ruchkin et al., 1988; McEvoy et al., 1998; Schupp et al., 2006b; Olofsson et al., 2008; Watts et al., 2014). In this systematic review, Kishiyama et al. (2009) found no significant differences in SW amplitudes between children from low-and high-SES groups. However, Czernochowski et al. (2008) observed larger SW amplitudes for high-SES compared to low-SES older adults using a recognition memory task.

In summary, this review found evidence that low-SES individuals exhibit lower ERP amplitudes than high-SES people for the following ERPs: P1, N1, N2, P3, ERN, Nd/PN, SW. These results are however not observed across all age groups. We found that a learning-related attenuation of the N400 was observed for high- but not for low-SES children, and studies using the Nd/PN provided evidence of heightened neural activity related to task-irrelevant stimuli for low-SES compared to high-SES individuals.

## Discussion

This systematic review found that a wide range of ERPs were significantly related to SES. With rare exceptions (notably the P2 component), we found reports of significant co-variations between SES and most ERPs of this systematic review (see Table 2; Figure 2). This finding has to be taken with caution as it is possible that null results went unpublished or unreported. Apparent contradictions between studies are another reason for caution. Further, most reported SES effects reflect a reduction of the amplitudes of specific ERPs for low-SES compared to high-SES individuals, which suggests that cognitive tasks elicit less neural activity in low- compared to high-SES individuals. Instances of heightened neural activity heightened neural activity for low-SES groups involved ERPs involved ERPs to distractor items, which suggests a greater difficulty to inhibit irrelevant information (D'Angiulli et al., 2008, 2012; Wray et al., 2017); or ERP activity reflecting a deficit in language learning (Ralph et al., 2020). These results are overall consistent with well-known behavioral results showing that high-SES individuals have a better performance than low-SES individuals on a wide array of cognitive tasks (e.g., Raizada and Kishiyama, 2010; Farah, 2017). Hereafter we discuss the implication of these findings, and we also propose avenues for future research in this field.

### SES and ERPs

We found SES effects on several ERPs with different spatiotemporal and functional properties (See Table 3; Figure 2). Effects involving the P3, slow waves and Nd/PN effects are consistent with the notion that EF is vulnerable to SES effects as these ERPs are often associated with controlled processes linked to EF, such as working memory updating, sustained maintenance of information in working memory and voluntary attention (Shallice, 1988; Miyake et al., 2000; Braver et al., 2001, 2007; Baddeley, 2006). In addition, SES effects on the N2 and ERN also suggest that early antecedents of the implementation of EF (Yeung et al., 2004) are also vulnerable to SES effects. These results may suggest that SES can predict neural activity related to different EF subprocesses. However, we also found SES effects involving ERPs that are not necessarily related to EF. For instance, SES effects on the N400 are compatible with results showing a consistent relationship between SES and language processes (Raizada and Kishiyama, 2010). It is important to point out that EF can be involved in many aspects of language (Merz et al., 2019) and thus it is not always possible to rule out that effects of SES on the N400 do not involve EF processes. Beyond language ERPs, a relationship between SES and very early ERPs such as the P1 and early Auditory sensory responses (ASR) indicate very strongly that SES effects on neural activity are not specific to EF-related neural systems. The P1 is a very early ERP linked to automatic attentional processes (Yago et al., 2004) and the very brief timing of ASRs also strongly suggests that very rapid automatic and basic sensory processes unrelated to controlled processes are also vulnerable to SES effects.

These results suggest that the relationship between SES and functional brain activity is not specific to a few neural systems. This would be consistent with results suggesting that SES predicts widespread differences in gray matter volume (Mackey et al., 2015). However, our results do not exclude the possibility that SES may have a more complex pattern of relationships with neural function, in which several different neural systems would be sensitive to SES effects and others not.

One of the main limitations of ERP studies focused on SES differences is that most of them have tested children, which leads to a scarcity of data on adults. This is a common bias in the field of research about how SES relates to cognition. Despite this shortcoming, we found in this review evidence suggesting potential developmental differences in how SES relates to ERP components. Specifically, we found that the P1, N1, and N2 were not related to SES for very early infants, whereas significant relationships were observed for older children. Similarly, slow waves (SW) were unaffected by SES in studies testing children, but SES predicted SW amplitude in a study with adults (Czernochowski et al., 2008), suggesting more protracted developmental effects. These results may tentatively suggest that SES effects on ERP activity can be observed only after some degree of neural development has been attained. However, this potential explanation does not account for more complex patterns of results such as the ones involving P3 amplitudes, in which SES effects were found for very young infants (Katus et al., 2020).

These results may point toward the possibility that the relationship between SES and ERP activity may interact with developmental stages, although this phenomenon may not be uniform across all ERP components. Further research is needed to explore this question, including studies focusing on teenagers and adults.

### Limitations and Future Research

Apart from the aforementioned bias toward studies focused on children, the field of studies examining SES effects on ERPs has a number of other limitations. First, there are unresolved contradictions which we detailed in the Results section. As discussed in the previous section, these contradictions may be linked to differences in the developmental stages of participants, but it may also be caused by differences in behavioral tasks used to elicit ERPs. This is particularly important for studies investigating the same ERPs with different behavioral tasks. For instance, Katus et al. (2020) and Kishiyama et al. (2009) have different results regarding the relationship between SES and early potentials (P1/N1/N2). Both studies used behavioral paradigms similar to an oddball task, but Katus et al. used auditory stimuli and Kishiyma et al. used visual stimuli. Another example of potential effects of task differences is visible in SW results where Czernochowski et al. (2008) observed SES effects using a task requiring recognition memory processes, whereas Kishyiama et al. did not observe SES effects on SW amplitude using an oddball-like task. These results tentatively suggest that beyond age differences, SES effects may also interact with the characteristics of the tasks used to elicit ERPs. For instance, critical task characteristics could include differences in the modality of the tasks (visual vs. auditory) and the nature of the processes involved in different tasks (e.g., recognition memory vs. selective attention). However, results involving the P3 ERP do not suggest any systematic task-related effects on the relationship between SES and ERP amplitudes. Specifically, similar SES effects on the P3 are observed when attentional tasks of different modalities were used (Katus et al., 2020; Wang and Yang, 2020) and differences in SES effects on the P3 were found when tasks tapping similar processes are used (Ruberry et al., 2017; St. John et al., 2019). Overall, this systematic review is unable to establish with certainty whether there are effects of behavioral task differences on the SES-ERP relationship. However, results from this review strongly suggest that future research will need to explore this question more systematically. Similarly, this review points toward the need to explore systematically the effects of electrode locations on how ERP components may be affected by SES. For instance, Katus et al. (2020) failed to observe effects of SES on the P1/N1 components using a midfrontal electrode, whereas Kishiyama et al. (2009) were able to observe SES effects on these potentials with posterior electrodes. However, The P3 was sensitive to SES both on frontal (Katus et al., 2020; Wang and Yang, 2020) and posterior electrodes (St. John et al., 2019).

Next, most reviewed studies have focused on testing the existences of an SES-ERP relationship, and ERP studies focusing on explanative models of how SES relates to cognition are rare. Skoe et al. (2013) provides a notable exception, as they report evidence that sensory stimulation plays a role in how SES relates to neural function. The future of research on SES-ERP relationships should focus on testing theoretical models of how SES predicts cognition, such as models focusing on cognitive stimulation (Rosen et al., 2019), models focusing on lifetime stress (Evans and Schamberg, 2009) and scarcity models (Mani et al., 2013). Other potential factors affecting both SES and ERPs should be considered too such as fatigue, sleep factors, level of nutrition, personality and psychopathology. More studies are also needed to test causal relationships between SES and ERP activity, such as longitudinal studies and experimental studies manipulating SES (Duncan and Magnuson, 2012).

All reviewed studies adopted a deficit approach of how SES predicts neural activity, as there was a focus on ERPs reflecting cognitive processes likely to be impaired for low-SES individuals. This approach is a common bias in this field of research, and it masks a promising and relatively unexplored avenue of research focusing on aspects of cognition that may be enhanced in people who live or grow up in poverty (Frankenhuis and Nettle, 2020). Finally, the majority of studies in this field have tested participants from WEIRD (Western, Educated, Industrialized, Rich and Democratic) countries (Henrich et al., 2010). This is problematic from the standpoint of generalization, and this is also counterintuitive as most people who live in poverty are living in non-WEIRD countries. Therefore, future research aiming to investigate how SES relates to neural function in non-WEIRD developing countries is urgently needed.

Overall, the current limitations of the field investigating SES-ERP relationships indicate that future studies will need to take into account different developmental stages; they will need to measure SES with multiple indicators (e.g., income, assets, and education); they will need to examine more systematically whether SES effects on specific ERPs interact with differences in task characteristics and electrode locations; they will need to consider more diverse samples; and they should also consider going beyond a deficit approach of how SES relates to cognitive and brain function.

### Conclusion

This systematic review found evidence that a broad range of ERPs were related to SES variations. These ERPs reflected different cognitive processes (e.g., automatic attention, overt attention, word learning, basic sensory processes, and executive function), which suggests that the relationship between SES and brain function is relatively homogeneous across different types of neural activity. A number of contradictory results were found, which could potentially be related to the different developmental stages of participants across studies. This review also found a number of limitations that point toward future research directions in this field: There is a bias toward studying children instead of adults, there is a bias toward samples from WEIRD countries; and there is a lack of ERP studies testing explanative models of how SES relates to cognition.

## Data Availability Statement

The original contributions presented in the study are included in the article/Supplementary Material, further inquiries can be directed to the corresponding author/s.

## Author Contributions

All authors participated in the manuscript preparation, including designing, writing, and revising the manuscript. HP-W.A. was involved in conceptualizing and designing data acquisition, writing of the manuscript, analysis, and interpretation. RK and KS did initial review. This was followed by critical revision by AS and final approval of the manuscript by KS.

## Conflict of Interest

The authors declare that the research was conducted in the absence of any commercial or financial relationships that could be construed as a potential conflict of interest.

## Footnotes

^1^Household typically refers to a group of people who live under the same roof.

^2^Throughout the manuscript, we refer to “Low-SES” and “High-SES” groups in terms of cognitive performance of differences in brain activity. It is important to highlight that these statements are based on central tendencies (averages of medians) calculated over a group of individuals. Therefore, it would be erroneous to infer from these statements that all individuals in these groups conform to the effects described in these statements. We thank one of our reviewers for highlighting this point.

## References

[B1] Adler N. E. Epel E. S. Castellazzo G. Ickovics J. R. (2000). Relationship of subjective and objective social status with psychological and physiological functioning: preliminary data in healthy white women. Health Psychol. 19, 586–592. 10.1037/0278-6133.19.6.586 11129362

[B2] Amso D. Salhi C. Badre D. (2018). The relationship between cognitive enrichment and cognitive control: a systematic investigation of environmental influences on development through socioeconomic status. Dev. Psychobiol. 61, 159–178. 10.1002/dev.21794 30375651 PMC8449852

[B3] Baddeley A. (2006). Working memory: an overview, in Working Memory and Education (San Diego, CA: Elsevier), 1–31. 10.1016/B978-012554465-8/50003-X

[B4] Bailey K. Mlynarczyk G. West R. (2016). Slow wave activity related to working memory maintenance in the N-back task. J. Psychophysiol. 30, 141–154. 10.1027/0269-8803/a000164

[B5] Bradley R. H. Corwyn R. F. Burchinal M. McAdoo H. P. García Coll C. (2001). The home environments of children in the United States Part II: relations with behavioral development through age thirteen. Child Dev. 72, 1868–1886. 10.1111/1467-8624.t01-1-00383 11768150

[B6] Brandolini A. Magri S. Smeeding T. M. (2010). Asset-based measurement of poverty. J. Policy Anal. Manage. 29, 267–284. 10.1002/pam.20491

[B7] Braver T. S. Barch D. M. Gray J. R. Molfese D. L. Snyder A. (2001). Anterior cingulate cortex and response conflict: effects of frequency, inhibition and errors. Cerebral Cortex 11, 825–836. 10.1093/cercor/11.9.825 11532888

[B8] Braver T. S. Gray J. R. Burgess G. C. (2007). Explaining the many varieties of working memory variation: dual mechanisms of cognitive control, in Variation in Working Memory (New York, NY: Oxford University Press), 76–106. 10.1093/acprof:oso/9780195168648.003.0004

[B9] Brooker R. J. (2018). Maternal behavior and socioeconomic status predict longitudinal changes in error-related negativity in preschoolers. Child Dev. 89, 725–733. 10.1111/cdev.13066 29611867 PMC5948123

[B10] Carretié L. Hinojosa J. A. Albert J. López-Martín S. De La Gándara B. S. Igoa J. M.. (2008). Modulation of ongoing cognitive processes by emotionally intense words. Psychophysiology 45, 188–196. 10.1111/j.1469-8986.2007.00617.x 17971056

[B11] Conejero Á. Guerra S. Abundis-Gutiérrez A. Rueda M. R. (2016). Frontal theta activation associated with error detection in toddlers: influence of familial socioeconomic status. Dev. Sci. 21:e12494. 10.1111/desc.12494 27981736

[B12] Czernochowski D. Fabiani M. Friedman D. (2008). Use it or lose it? SES mitigates age-related decline in a recency/recognition task. Neurobiol. Aging 29, 945–958. 10.1016/j.neurobiolaging.2006.12.017 17280741 PMC2440484

[B13] D'Angiulli A. Herdman A. Stapells D. Hertzman C. (2008). Children's event-related potentials of auditory selective attention vary with their socioeconomic status. Neuropsychology 22, 293–300. 10.1037/0894-4105.22.3.293 18444707

[B14] D'Angiulli A. van Roon P. Weinberg J. Oberlander T. F. Grunau R. E. Hertzman C.. (2012). Frontal EEG/ERP correlates of attentional processes, cortisol and motivational states in adolescents from lower and higher socioeconomic status. Front. Hum. Neurosci. 6:306. 10.3389/fnhum.2012.00306 23181016 PMC3500742

[B15] Dehaene S. Posner M. I. Tucker D. M. (1994). Localization of a neural system for error detection and compensation. Psychol. Scie. 5, 303–305. 10.1111/j.1467-9280.1994.tb00630.x

[B16] Donchin E. (1981). Surprise!…Surprise? Psychophysiology 18, 493–513. 10.1111/j.1469-8986.1981.tb01815.x 7280146

[B17] Duncan G. J. Magnuson K. (2012). Socioeconomic status and cognitive functioning: moving from correlation to causation. Wiley Interdiscip. Rev. Cogn. Sci. 3, 377–386. 10.1002/wcs.1176 26301469

[B18] Duncan G. J. Magnuson K. Kalil A. Ziol-Guest K. (2012). The importance of early childhood poverty. Soc. Indic. Res. 108, 87–98. 10.1007/s11205-011-9867-9

[B19] Eimer M. (2000). Event-related brain potentials distinguish processing stages involved in face perception and recognition. Clin. Neurophysiol. 111, 694–705. 10.1016/S1388-2457(99)00285-0 10727921

[B20] Evans G. W. Kim P. (2007). Childhood poverty and health: cumulative risk exposure and stress dysregulation. Psychol. Sci. 18, 953–957. 10.1111/j.1467-9280.2007.02008.x 17958708

[B21] Evans G. W. Schamberg M. A. (2009). Childhood poverty, chronic stress, and adult working memory. Proc. Natl. Acad. Sci. U.S.A. 106, 6545–6549. 10.1073/pnas.0811910106 19332779 PMC2662958

[B22] Farah M. J. (2017). The neuroscience of socioeconomic status: correlates, causes, and consequences. Neuron 96, 56–71. 10.1016/j.neuron.2017.08.034 28957676

[B23] Farah M. J. (2018). Socioeconomic status and the brain: prospects for neuroscience-informed policy. Nat. Rev. Neurosci. 19, 428–438. 10.1038/s41583-018-0023-2 29867123

[B24] Farah M. J. Shera D. M. Savage J. H. Betancourt L. Giannetta J. M. Brodsky N. L.. (2006). Childhood poverty: specific associations with neurocognitive development. Brain Res. 1110, 166–174. 10.1016/j.brainres.2006.06.072 16879809

[B25] Fernald A. Marchman V. A. Weisleder A. (2013). SES differences in language processing skill and vocabulary are evident at 18 months. Dev. Sci. 16, 234–248. 10.1111/desc.12019 23432833 PMC3582035

[B26] Fernald L. C. H. Weber A. Galasso E. Ratsifandrihamanana L. (2011). Socioeconomic gradients and child development in a very low income population: evidence from Madagascar. Dev. Sci. 14, 832–847. 10.1111/j.1467-7687.2010.01032.x 21676102

[B27] Fernández T. Silva-Pereyra J. Prieto-Corona B. Rodríguez-Camacho M. Reynoso-Alcántara V. (2014). Event-related brain potentials during a semantic priming task in children with learning disabilities not otherwise specified. PLoS ONE 9:e105318. 10.1371/journal.pone.0105318 25144188 PMC4140769

[B28] Folstein J. R. Van Petten C. (2008). Influence of cognitive control and mismatch on the N2 component of the ERP: a review. Psychophysiology 45, 152–170. 10.1111/j.1469-8986.2007.00602.x 17850238 PMC2365910

[B29] Frankenhuis W. E. Nettle D. (2020). The strengths of people in poverty. Curr. Directions Psychol. Sci. 29, 16–21. 10.1177/0963721419881154

[B30] Friedman N. P. Miyake A. (2017). Unity and diversity of executive functions: individual differences as a window on cognitive structure. Cortex 86, 186–204. 10.1016/j.cortex.2016.04.023 27251123 PMC5104682

[B31] Galobardes B. Shaw M. Lawlor D. A. Lynch J. W. Smith G. D. (2006). Indicators of socioeconomic position (part 1). J. Epidemiol. Community Health 60, 7–12. 10.1136/jech.2004.023531 16361448 PMC2465546

[B32] García-Larrea L. Cézanne-Bert G. (1998). P3, Positive slow wave and working memory load: a study on the functional correlates of slow wave activity. Electroencephalogr. Clin. Neurophysiol. 108, 260–273. 10.1016/S0168-5597(97)00085-3 9607515

[B33] Gehring W. J. Goss B. Coles M. G. H. Meyer D. E. Donchin E. (2018). The error-related negativity. Perspect. Psychol. Sci. 13, 200–204. 10.1177/1745691617715310 29592655

[B34] Gehring W. J. Goss B. Coles M. G. H. H. Meyer D. E. Donchin E. (1993). A neural system for error detection and compensation. Psychol. Sci. 4, 385–390. 10.1111/j.1467-9280.1993.tb00586.x

[B35] Giuliano R. J. Karns C. M. Roos L. E. Bell T. A. Petersen S. Skowron E. A.. (2018). Effects of early adversity on neural mechanisms of distractor suppression are mediated by sympathetic nervous system activity in preschool-aged children. Dev. Psychol. 54, 1674–1686. 10.1037/dev0000499 30148395 PMC12614223

[B36] Goto N. Mushtaq F. Shee D. Lim X. L. Mortazavi M. Watabe M.. (2017). Neural signals of selective attention are modulated by subjective preferences and buying decisions in a virtual shopping task. Biol. Psychol. 128, 11–20. 10.1016/j.biopsycho.2017.06.004 28666891

[B37] Hackman D. A. Farah M. J. (2009). Socioeconomic status and the developing brain. Trends Cognitive Sci. 13, 65–73. 10.1016/j.tics.2008.11.003 19135405 PMC3575682

[B38] Hackman D. A. Farah M. J. Meaney M. J. (2010). Socioeconomic status and the brain: Mechanistic insights from human and animal research. Nat. Rev. Neurosci. 11, 651–659. 10.1038/nrn2897 20725096 PMC2950073

[B39] Hackman D. A. Gallop R. Evans G. W. Farah M. J. (2015). Socioeconomic status and executive function: developmental trajectories and mediation. Dev. Sci. 18, 686–702. 10.1111/desc.12246 25659838

[B40] Hair N. L. Hanson J. L. Wolfe B. L. Pollak S. D. (2015). Association of child poverty, brain development, and academic achievement. JAMA Pediatrics 169, 822–829. 10.1001/jamapediatrics.2015.1475 26192216 PMC4687959

[B41] Haushofer J. Fehr E. (2014). On the psychology of poverty. Science 344, 862–867. 10.1126/science.1232491 24855262

[B42] Heidlmayr K. Kihlstedt M. Isel F. (2020). A review on the electroencephalography markers of Stroop executive control processes. Brain Cognition 146:105637. 10.1016/j.bandc.2020.105637 33217721

[B43] Henrich J. Heine S. J. Norenzayan A. (2010). The weirdest people in the world? Behav. Brain Sci. 33, 61–83. 10.1017/S0140525X0999152X 20550733

[B44] Hillyard S. A. Hink R. F. Schwent V. L. Picton T. W. (1973). Electrical signs of selective attention in the human brain. Science 182, 177–180. 10.1126/science.182.4108.177 4730062

[B45] Hoff-Ginsberg E. (1998). The relation of birth order and socioeconomic status to children's language experience and language development. Appl. Psycholinguistics 19, 603–629. 10.1017/S0142716400010389

[B46] Holroyd C. B. Coles M. G. H. (2002). The neural basis of human error processing: reinforcement learning, dopamine, and the error-related negativity. Psychol. Rev. 109, 679–709. 10.1037/0033-295X.109.4.679 12374324

[B47] Isbell E. Wray A. H. Neville H. J. (2016). Individual differences in neural mechanisms of selective auditory attention in preschoolers from lower socioeconomic status backgrounds: an event-related potentials study. Dev. Sci. 19, 865–880. 10.1111/desc.12334 26234822

[B48] Kaplan G. A. Turrell G. Lynch J. W. Everson S. A. Helkala E. L. Salonen J. T. (2001). Childhood socioeconomic position and cognitive function in adulthood. Int. J. Epidemiol. 30, 256–263. 10.1093/ije/30.2.256 11369724

[B49] Katus L. Mason L. Milosavljevic B. McCann S. Rozhko M. Moore S. E.. (2020). ERP markers are associated with neurodevelopmental outcomes in 1–5 month old infants in rural Africa and the UK. NeuroImage 210:116591. 10.1016/j.neuroimage.2020.116591 32007497 PMC7068721

[B50] Kim P. Evans G. W. Angstadt M. Ho S. S. Sripada C. S. Swain J. E.. (2013). Effects of childhood poverty and chronic stress on emotion regulatory brain function in adulthood. Proc. Natl. Acad. Sci. U.S.A. 110, 18442–18447. 10.1073/pnas.1308240110 24145409 PMC3831978

[B51] Kishiyama M. M. Boyce W. T. Jimenez A. M. Perry L. M. Knight R. T. (2009). Socioeconomic disparities affect prefrontal function in children. J. Cognitive Neurosci. 21, 1106–1115. 10.1162/jocn.2009.21101 18752394

[B52] Kornhauser A. W. (1918). The economic standing of parents and the intelligence of their children. J. Educ. Psychol. 9, 159–164. 10.1037/h0072624

[B53] Korpilahti P. Valkama M. Jansson-Verkasalo E. (2017). Event-related potentials reflect deficits in lexical access: the N200 in prematurely born school-aged children. Folia Phoniatrica et Logopaedica 68, 189–198. 10.1159/000450886 28253505

[B54] Kutas M. Federmeier K. D. (2011). Thirty years and counting: finding meaning in the N400 component of the Event-Related Brain Potential (ERP). Annual Rev. Psychol. 62, 621–647. 10.1146/annurev.psych.093008.131123 20809790 PMC4052444

[B55] Last B. S. Lawson G. M. Breiner K. Steinberg L. Farah M. J. (2018). Childhood socioeconomic status and executive function in childhood and beyond. PLoS ONE 13:e0202964. 10.1371/journal.pone.0202964 30142188 PMC6108482

[B56] Lawson G. M. Hook C. J. Farah M. J. (2018). A meta-analysis of the relationship between socioeconomic status and executive function performance among children. Dev. Sci. 21:e12529. 10.1111/desc.12529 28557154 PMC5821589

[B57] Leonard J. A. Romeo R. R. Park A. T. Takada M. E. Robinson S. T. Grotzinger H.. (2019). Associations between cortical thickness and reasoning differ by socioeconomic status in development. Dev. Cogn. Neurosci. 36:100641. 10.1016/j.dcn.2019.100641 30951970 PMC6969225

[B58] Loeb E. Hurd N. M. (2019). Subjective social status, perceived academic competence, and academic achievement among underrepresented students. J. College Stud. Retention Res. Theory Pract. 21, 150–165. 10.1177/1521025117696821

[B59] Luck S. J. (2005). An Introduction to the Event-Related Potential Technique. Cambridge, MA: MIT Press.

[B60] Luck S. J. Kappenman E. (2011). The Oxford Handbook of Event-Related Potential Components. New York, NY: Oxford University Press 10.1093/oxfordhb/9780195374148.001.0001

[B61] Mackey A. P. Finn A. S. Leonard J. A. Jacoby-Senghor D. S. West M. R. Gabrieli C. F. O.. (2015). Neuroanatomical correlates of the income-achievement gap. Psychol. Sci. 26, 925–933. 10.1177/0956797615572233 25896418 PMC4458190

[B62] Mani A. Mullainathan S. Shafir E. Zhao J. (2013). Poverty impedes cognitive function. Science 341, 976–980. 10.1126/science.1238041 23990553

[B63] McEvoy L. K. Smith M. E. Gevins A. (1998). Dynamic cortical networks of verbal and spatial working memory: effects of memory load and task practice. Cerebral Cortex 8, 563–574. 10.1093/cercor/8.7.563 9823478

[B64] McLoyd V. C. (1998). Socioeconomic disadvantage and child development. Am. Psychol. 53, 185–204. 10.1037/0003-066X.53.2.185 9491747

[B65] Merz E. C. Wiltshire C. A. Noble K. G. (2019). Socioeconomic inequality and the developing brain: spotlight on language and executive function. Child Dev. Perspect. 13, 15–20. 10.1111/cdep.12305

[B66] Mestres-Missé A. Rodriguez-Fornells A. Münte T. F. (2007). Watching the brain during meaning acquisition. Cerebral Cortex 17, 1858–1866. 10.1093/cercor/bhl094 17056648

[B67] Mezzacappa E. (2004). Alerting, orienting, and executive attention: developmental properties and sociodemographic correlates in an epidemiological sample of young, urban children. Child Dev. 75, 1373–1386. 10.1111/j.1467-8624.2004.00746.x 15369520

[B68] Miyake A. Friedman N. P. Emerson M. J. Witzki A. H. Howerter A. Wager T. D. (2000). The unity and diversity of executive functions and their contributions to complex “frontal lobe” tasks: a latent variable analysis. Cognitive Psychol. 41, 49–100. 10.1006/cogp.1999.0734 10945922

[B69] Moher D. Liberati A. Tetzlaff J. Altman D. G. (2010). Preferred reporting items for systematic reviews and meta-analyses: the PRISMA statement. Int. J. Surg. 8, 336–341. 10.1016/j.ijsu.2010.02.007 20171303

[B70] Mueller V. Brehmer Y. von Oertzen T. Li S.-C. Lindenberger U. (2008). Electrophysiological correlates of selective attention: a lifespan comparison. BMC Neurosci. 9:18. 10.1186/1471-2202-9-18 18237433 PMC2270855

[B71] Mushtaq F. Bland A. R. Schaefer A. (2011). Uncertainty and cognitive control. Front. Psychol. 2:249. 10.3389/fpsyg.2011.00249 22007181 PMC3184613

[B72] Noble K. G. Houston S. M. Brito N. H. Bartsch H. Kan E. Kuperman J. M.. (2015). Family income, parental education and brain structure in children and adolescents. Nat. Neurosci. 18, 773–778. 10.1038/nn.3983 25821911 PMC4414816

[B73] Noble K. G. Wolmetz M. E. Ochs L. G. Farah M. J. McCandliss B. D. (2006). Brain-behavior relationships in reading acquisition are modulated by socioeconomic factors. Dev. Sci. 9, 642–654. 10.1111/j.1467-7687.2006.00542.x 17059461

[B74] Olofsson J. K. Nordin S. Sequeira H. Polich J. (2008). Affective picture processing: an integrative review of ERP findings. Biol. Psychol. 77, 247–265. 10.1016/j.biopsycho.2007.11.006 18164800 PMC2443061

[B75] Ong Q. Theseira W. Ng I. Y. H. (2019). Reducing debt improves psychological functioning and changes decision-making in the poor. Proc. Natl. Acad. Sci. U.S.A. 116, 7244–7249. 10.1073/pnas.1810901116 30910964 PMC6462060

[B76] Pavlov Y. G. Adamian N. Appelhoff S. Arvaneh M. Benwell C. Beste C. (2020). EEGManyLabs: investigating the replicability of influential EEG experiments. PsyArxiv [Preprint]. 10.31234/osf.io/528nr 33965167

[B77] Perkins S. Finegood E. Swain J. (2013). Poverty and language development: roles of parenting and stress. Innovations Clin. Neurosci. 10, 10–19. 23696954 PMC3659033

[B78] Picton T. W. Hillyard S. A. (1974). Human auditory evoked potentials. II: effects of attention. Electroencephalogr. Clin. Neurophysiol. 36, 191–200. 10.1016/0013-4694(74)90156-4 4129631

[B79] Polich J. (2007). Updating P300: an integrative theory of P3a and P3b. Clin. Neurophysiol. 118, 2128–2148. 10.1016/j.clinph.2007.04.019 17573239 PMC2715154

[B80] Raizada R. D. S. Kishiyama M. M. (2010). Effects of socioeconomic status on brain development, and how cognitive neuroscience may contribute to levelling the playing field. Front. Hum. Neurosci. 4:3. 10.3389/neuro.09.003.2010 20161995 PMC2820392

[B81] Ralph Y. K. Schneider J. M. Abel A. D. Maguire M. J. (2020). Using the N400 event-related potential to study word learning from context in children from low- and higher-socioeconomic status homes. J. Exp. Child Psychol. 191:104758. 10.1016/j.jecp.2019.104758 31855830 PMC8191850

[B82] Revonsuo A. Laine M. (1996). Semantic processing without conscious understanding in a global aphasic: evidence from auditory event-related brain potentials. Cortex 32, 29–48. 10.1016/S0010-9452(96)80015-3 8697750

[B83] Rosen M. L. Hagen M. P. Lurie L. A. Miles Z. E. Sheridan M. A. Meltzoff A. N.. (2019). Cognitive stimulation as a mechanism linking socioeconomic status with executive function: a longitudinal investigation. Child Dev. 91, e762–e779. 10.1111/cdev.13315 31591711 PMC7138720

[B84] Rosen M. L. Sheridan M. A. Sambrook K. A. Meltzoff A. N. McLaughlin K. A. (2018). Socioeconomic disparities in academic achievement: a multi-modal investigation of neural mechanisms in children and adolescents. NeuroImage 173, 298–310. 10.1016/j.neuroimage.2018.02.043 29486324 PMC5944356

[B85] Rowe M. L. Goldin-Meadow S. (2009). Early gesture selectively predicts later language learning. Dev. Sci. 12, 182–187. 10.1111/j.1467-7687.2008.00764.x 19120426 PMC2677374

[B86] Roy A. L. McCoy D. C. Cybele Raver C. (2014). Instability versus quality: residential mobility, neighborhood poverty, and children's self-regulation. Dev. Psychol. 50, 1891–1896. 10.1037/a0036984 24842459 PMC4727396

[B87] Ruberry E. J. Lengua L. J. Crocker L. H. Bruce J. Upshaw M. B. Sommerville J. A. (2017). Income, neural executive processes, and preschool children's executive control. Dev. Psychopathol. 29, 143–154. 10.1017/S095457941600002X 26817409

[B88] Ruchensky J. R. Bauer E. A. MacNamara A. (2020). Intolerance of uncertainty, depression and the error-related negativity. Int. J. Psychophysiol. 153, 45–52. 10.1016/j.ijpsycho.2020.04.015 32330538 PMC7934182

[B89] Ruchkin D. S. Johnson R. Canoune H. Ritter W. (1990). Short-term memory storage and retention: an event-related brain potential study. Electroencephalogr. Clin. Neurophysiol. 76, 419–439. 10.1016/0013-4694(90)90096-3 1699736

[B90] Ruchkin D. S. Johnson R. Mahaffey D. Sutton S. (1988). Toward a functional categorization of slow waves. Psychophysiology 25, 339–353. 10.1111/j.1469-8986.1988.tb01253.x 3406333

[B91] Ruge H. Braver T. S. (2007). Neural mechanisms of cognitive control in cued task-switching: rules, representations, and preparation, in Neuroscience of Rule-Guided Behavior (New York, NY). 255–282 10.1093/acprof:oso/9780195314274.003.0015

[B92] Schaefer A. Pottage C. L. Rickart A. J. (2011). Electrophysiological correlates of remembering emotional pictures. NeuroImage 54, 714–724. 10.1016/j.neuroimage.2010.07.030 20650320

[B93] Schupp H. T. Flaisch T. Stockburger J. Junghöfer M. (2006a). Emotion and attention: event-related brain potential studies. Prog. Brain Res. 156, 31–51. 10.1016/S0079-6123(06)56002-9 17015073

[B94] Schupp H. T. Stockburger J. Codispoti M. Junghöfer M. Weike A. I. Hamm A. O. (2006b). Stimulus novelty and emotion perception: the near absence of habituation in the visual cortex. NeuroReport 17, 365–369. 10.1097/01.wnr.0000203355.88061.c6 16514360

[B95] Shah A. K. Mullainathan S. Shafir E. (2012). Some consequences of having too little. Science 338, 682–685. 10.1126/science.1222426 23118192

[B96] Shallice T. (1988). From Neuropsychology to Mental Structure. New York, NY: Cambridge University Press 10.1017/CBO9780511526817

[B97] Skoe E. Kraus N. (2010). Auditory brain stem response to complex sounds: a tutorial. Ear Hearing 31, 302–324. 10.1097/AUD.0b013e3181cdb272 20084007 PMC2868335

[B98] Skoe E. Krizman J. Kraus N. (2013). The impoverished brain: disparities in maternal education affect the neural response to sound. J. Neurosci. 33, 17221–17231. 10.1523/JNEUROSCI.2102-13.2013 24174656 PMC6618371

[B99] Smart E. L. Gow A. J. Deary I. J. (2014). Occupational complexity and lifetime cognitive abilities. Neurology 83, 2285–2291. 10.1212/WNL.0000000000001075 25411439 PMC4277669

[B100] Starr A. Aguinaldo T. Roe M. Michalewski H. J. (1997). Sequential changes of auditory processing during target detection: motor responding versus mental counting. Electroencephalogr. Clin. Neurophysiol. 105, 201–212. 10.1016/S0924-980X(97)00016-7 9216489

[B101] St. John A. M. Finch K. Tarullo A. R. (2019). Socioeconomic status and neural processing of a go/no-go task in preschoolers: an assessment of the P3b. Dev. Cognitive Neurosci. 38:100677. 10.1016/j.dcn.2019.100677 31255904 PMC6969333

[B102] Tacconelli E. (2010). Systematic reviews: CRD's guidance for undertaking reviews in health care. In Lancet Infect. Dis. 10:P226 10.1016/S1473-3099(10)70065-7

[B103] Taylor J. B. Visser T. A. W. Fueggle S. N. Bellgrove M. A. Fox A. M. (2018). The error-related negativity (ERN) is an electrophysiological marker of motor impulsiveness on the Barratt Impulsiveness Scale (BIS-11) during adolescence. Dev. Cognitive Neurosci. 30, 77–86. 10.1016/j.dcn.2018.01.003 29353681 PMC6969191

[B104] The World Bank (2020). Poverty Overview. Retrieved from: https://www.worldbank.org/en/topic/poverty/overview

[B105] Tine M. (2014). Working memory differences between children living in rural and urban poverty. J. Cognition Dev. 15, 599–613. 10.1080/15248372.2013.797906 25554726 PMC4263265

[B106] Van Veen V. Carter C. S. (2002). The anterior cingulate as a conflict monitor: FMRI and ERP studies. Physiol. Behav. 77, 477–482. 10.1016/S0031-9384(02)00930-7 12526986

[B107] Walker S. O'Connor D. B. Schaefer A. (2011). Brain potentials to emotional pictures are modulated by alexithymia during emotion regulation. Cognitive Affect. Behav. Neurosci. 11, 463–475. 10.3758/s13415-011-0042-1 21614450

[B108] Wang S. Yang D. (2020). The effects of poverty stereotype threat on inhibition ability in individuals from different income-level families. Brain Behav. 10:e01770. 10.1002/brb3.1770 33089971 PMC7749600

[B109] Watts S. Buratto L. G. Brotherhood E. V. Barnacle G. E. Schaefer A. (2014). The neural fate of neutral information in emotion-enhanced memory. Psychophysiology 51, 673–684. 10.1111/psyp.12211 24673606

[B110] Weinberg A. Klein D. N. Hajcak G. (2012). Increased error-related brain activity distinguishes generalized anxiety disorder with and without comorbid major depressive disorder. J. Abnormal Psychol. 121, 885–896. 10.1037/a0028270 PMC3659236 22564180

[B111] Wicherts J. M. Scholten A. Z. (2013). Comment on “Poverty Impedes Cognitive Function.” Science 342, 1169–1169. 10.1126/science.1246680 24311665

[B112] Wray A. H. Stevens C. Pakulak E. Isbell E. Bell T. Neville H. (2017). Development of selective attention in preschool-age children from lower socioeconomic status backgrounds. Dev. Cognitive Neurosci. 26, 101–111. 10.1016/j.dcn.2017.06.006 28735165 PMC5703215

[B113] Yago E. Duarte A. Wong T. Barceló F. Knight R. T. (2004). Temporal kinetics of prefrontal modulation of the extrastriate cortex during visual attention. Cognitive Affect. Behav. Neurosci. 4, 609–617. 10.3758/CABN.4.4.609 15849901

[B114] Yeung N. Botvinick M. M. Cohen J. D. (2004). The neural basis of error detection: conflict monitoring and the error-related negativity. Psychol. Rev. 111, 931–959. 10.1037/0033-295X.111.4.931 15482068

[B115] Yong M. H. Lim X. L. Schaefer A. (2020). How do Asians perceive caucasian eyes? Electrophysiological correlates of perceiving racial differences from the eyes region of the face. Neurosci. Letters 720:134759. 10.1016/j.neulet.2020.134759 31952988

