# Supplementary material for: The Relationship Between Socioeconomic Status and Scalp Event-Related Potentials: A Systematic Review
Source: Front Hum Neurosci. 2021 Jan 27;15:601489. doi: 10.3389/fnhum.2021.601489 (PMC7873529; doi:10.3389/fnhum.2021.601489)
Supplement: Supplementary file 1 [file Table_1.DOCX]

Supplementary Material

Supplementary Table 1

| **Article** | **Offline reference site** | **Mean number of artifact free trials used for averaging** |
| --- | --- | --- |
| Katus et al. (2020) | Unilateral left mastoid was used during data collection. However, no re-referencing was done during offline analysis. | UK cohort:  At 1-month = 61.36  At 5-months = 52.72  Gambian cohort:  At 1-month = 61.95  At 5-months = 52.58  (Across all three conditions) |
| Ralph et al. (2020) | Average scalp reference. | At least 24 trials (9 per condition) |
| Wray et al. (2017) | Mean mastoid | High-SES  Attended = 232  Unattended = 237  Low-SES, Y1 (Y2)  Attended = 259 (289)  Unattended = 258 (286)  Out of 400 |
| Kishiyama et al. (2009) | Left and right earlobes | Not mentioned |
| D’Angiulli et al. (2008) | Nose tip | Not mentioned |
| D’Angiulli et al. (2012) | Average scalp reference | Not mentioned |
| St. John et al. (2019) | Average scalp reference | Go trials = 63.45/210  No-go trials = 24.59/70 |
| Ruberry et al. (2017) | Cz vertex electrode | Frog-fish task:  Minimum 120/240 correct trials  Flanker task:  Minimum 50/100 correct trials |
| Giuliano et al. (2018) | Mean mastoid | Not mentioned |
| Skoe et al. (2013) | Right earlobe | Average of 3000 trials |
| Czernochowski et al. (2008) | Nose tip | Young participants:  Recency trials =33/60  Recognition trials = 44/60  Old high-SES participants:  Recency trials =21/60  Recognition trials = 31/60  Old low-SES participants:  Recency trials =20/60  Recognition trials = 32/60 |
| Conejero et al. (2016) | Average scalp reference | Mean valid trials per condition was 8.15 (correct), 8.69 (position error) and 8.57 (conceptual error) out of 12 trails per condition |
| Brooker (2018) | Mean mastoid | Not mentioned |
| Wang & Yang (2020) | Mean mastoid | Not mentioned |
